# Pesticide residues on milkweed and strawberry at small farms and non-target effects of two fungicides on monarch butterfly caterpillars

**DOI:** 10.7717/peerj.20729

**Published:** 2026-02-10

**Authors:** Amy P. Hastings, Van Hniang Par, Scott H. McArt, Anurag A. Agrawal

**Affiliations:** 1 Department of Ecology and Evolutionary Biology, Cornell University, Ithaca, NY, United States of America; 2 Department of Entomology, Cornell University, Ithaca, NY, United States of America

**Keywords:** Fungicide, Insect declines, Milkweed, Monarch butterfly, Non-target effects, Pesticide residues, Plant–insect interactions, Strawberry

## Abstract

Concern over insect declines has increased attention on the effects of pesticide residues on native insects. We collected strawberry (target) and common milkweed (non-target) foliage and flowers on two small Central New York farms, within crop fields as well as field margins, and analyzed the tissues for pesticide residues of 94 agrochemicals. We found quantifiable levels of 13 fungicides, herbicides, and insecticides, mostly at low concentrations (typically less than 200 ppb where detected), and more often on strawberry than milkweed. We generally found higher pesticide residue levels early in the season (June *vs.* July) and on leaves compared to flowers. Residue levels in fields did not differ strongly from margins but pesticide drift may have left low-level residues on milkweed leaves and flowers in margins. Given that non-target effects of fungicides are understudied, we selected two prevalent fungicides found in this study (cyprodinil and difenoconazole) and used them in laboratory assays to assess impacts on early instar monarch (*Danaus plexippus*) caterpillar feeding, growth, and growth efficiency on common milkweed, along with two other milkweed (*Asclepias*) species. We tested the hypothesis that exposure to pesticides may be most impactful on poor quality host plants. For both fungicides, exposure at the highest doses (>100,000 ppb) reduced feeding, with the strongest effect on *Asclepias asperula*, the lowest quality host plant. Effects on caterpillar growth were similarly negative and consistent across host plant species. Finally, effects of fungicides on gross growth efficiency of caterpillars were negative, but dependent on the fungicide. Effects of cyprodinil were stronger than difenoconazole, but at realistically low concentrations there was little effect of either fungicide. Nonetheless, higher concentrations of these chemicals, approximating those experienced directly after fungicide application, may impact non-target species. The observed interaction of fungicides with host plant species highlights the importance of considering resource quality in the assessment of non-target effects of pesticides.

## Introduction

There is widespread evidence of a worldwide decline in insects, both at the level of species loss as well as decreases in abundance (Dirzo et al., 2014; Goulson et al., 2015; Vogel, 2017; Hallmann et al., 2017; Warren et al., 2021). Given the important roles insects play in linking primary producers to consumers, decomposition and nutrient cycling, pollination of natural and agricultural plants, and biological control of agricultural pests, it is critical that we work to understand the causes of their declines (Wagner et al., 2021). Along with climate change and habitat loss, intensified agricultural practices have been identified as an important contributor to insect declines, especially the adoption of largescale application of pesticides (Ollerton et al., 2014; Sánchez-Bayo & Wyckhuys, 2019; Wagner et al., 2021).

Among agrochemicals, insecticides, by definition, likely pose the greatest hazard to non-target insects. Broad-spectrum and systemic insecticides, of which neonicotinoids have received the most attention, have major effects, including reduced survival of soil and aquatic invertebrates, acute and chronic lethal and sublethal effects on honeybees and bumblebees, and are associated with community-wide declines in butterfly abundance and richness (Van Lexmond et al., 2015; Pisa et al., 2015; Mulé et al., 2017; Tosi, Burgio & Nieh, 2017; Deynze et al., 2024; Basley & Goulson, 2018). On top of these direct effects of insecticides, the broadscale use of herbicides in modern agriculture has introduced negative indirect effects on insect populations by reducing plant diversity in agricultural fields, thus reducing the survival of insects that rely on interactions with wild plants (Hyvönen & Salonen, 2002; Egan et al., 2014; Sánchez-Bayo & Wyckhuys, 2019; Mallick, Rana & Ghosh, 2023).

Less well studied are fungicides, which can dominate the set of pesticide residues found in insect samples (McArt et al., 2017; Rondeau & Raine, 2022; Bischoff, Baert & McArt, 2023; Mueller et al., 2024). Some fungicides are directly toxic to insects (Amarasekare & Shearer, 2013; Cornejo et al., 2020; Halsch et al., 2022; De Souza et al., 2024; Riihimäki et al., 2025), while others are known to act synergistically with insecticides in killing insects (Iverson et al., 2019; Schuhmann et al., 2022). Recent advances using mass spectrometry have enabled higher throughput and increased sensitivity in the detection of pesticide residues, improving our understanding of risks to non-target organisms at multiple scales (Mitchell et al., 2017; Brühl et al., 2021; Bischoff, Baert & McArt, 2023; David et al., 2016; Runquist, Nordmeyer & Stapleton, 2024).

Among insects, the monarch butterfly (*Danaus plexippus*) has been an iconic sentinel in the discussion of insect declines (Losey, Rayor & Carter, 1999; Oberhauser & Solensky, 2004; Pleasants et al., 2015; Gustafsson et al., 2015; Zylstra et al., 2021). Monarch populations have declined by approximately 75% over the past three decades (Brower et al., 2012; Thogmartin et al., 2017; Shirey & Ries, 2023) and there is a litany of proposed causes, including climate change, overwintering habitat loss due to logging, insecticides, and loss of milkweed, as reviewed in Agrawal (2017). A major driver of milkweed loss in monarch summer breeding regions is thought to be intensified agricultural practices that use herbicides (especially glyphosate), removing milkweeds from crop fields and edges, where it grows readily (Hartzler & Buhler, 2000; Oberhauser et al., 2001; Stenoien et al., 2018; Martin et al., 2021). Recent analyses have identified residues of dozens of insecticides, herbicides, and fungicides on milkweed plants in agricultural fields, urban landscapes, and open habitats of the Midwest and California (Olaya-Arenas & Kaplan, 2019; Halsch et al., 2020), and experimental studies have demonstrated lethal (Krishnan et al., 2021) and sublethal effects of some insecticides (Prouty et al., 2021; Prouty et al., 2023; Krishnan et al., 2021) and fungicides (Olaya-Arenas et al., 2020) on monarchs exposed to pesticides at different life-stages. These studies suggest that monarchs may be at risk of toxic effects from agrochemicals encountered in their summer breeding grounds, although there is evidence that they may be able to detect and avoid some chemicals (Olaya-Arenas, Scharf & Kaplan, 2020).

Given the propensity of common milkweed (*Asclepias syriaca*) to grow among crops in eastern North America, especially in and around farms that do not heavily use pesticides or other chemicals, we collected tissues from strawberry and milkweed plants from two small strawberry farms in central New York State to test for residues of up to 94 pesticides. These farms are typical of the region for u-pick strawberries and are prime habitat for where monarch butterflies forage in the summer months. When pesticides are applied on such small farms, they are typically applied early in the growing season using boom sprayers mounted to tractors. Levels of pesticide residues on these farms should provide underestimates of what insects encounter on their hosts in more conventional agricultural areas where pesticide use is typically much higher (Graham, McArt & Isaacs, 2024). For the fungicides relevant to this study commonly employed in New York State, field residue levels have been found up to 2,000 ppb (Frazier et al., 2015; McArt et al., 2017; Mueller et al., 2024), although most are well under 1,000 ppb (Graham et al., 2021; Rondeau et al., 2022; Siviter et al., 2023); accordingly, we designate values under 200 ppb as “low”. However, even low concentrations of fungicide residues can have sublethal effects on non-target organisms (less than 10 ppb in Olaya-Arenas et al., 2020). In our survey, we expected to find greater levels of pesticide residues on the strawberry plants than on non-target milkweeds, early in the growing season compared to late in the season, on milkweed leaves compared to flowers, and in crop fields compared to margins.

In the laboratory, we tested for direct effects of the two most abundant fungicides we identified in the field, cyprodinil and difenoconazole. We measured monarch caterpillar survival and growth across a range of fungicide doses on three milkweed hosts: *Asclepias syriaca*—the dominant host plant in eastern North America, *Asclepias curassavica*—an ornamental milkweed which has naturalized in the southern USA, and *Asclepias asperula*—an important, yet highly toxic, host plant species to the first spring generation of monarchs in the southern USA (Agrawal, 2017). Both *A. curassavica* and *A. asperula* grow near farms in the southern US, and these species also contain higher levels of plant toxins than *A. syriaca*. Accordingly, we hypothesized that caterpillars would survive well on all three milkweed species, but that caterpillars would grow at different rates, and that there would be increasing negative effects of the fungicides on monarch performance on the poorer quality host plant species. In this case, *A. asperula* is likely the poorest host, as it is characterized by the highest levels of latex exudation (a potent defense; see Agrawal & Konno, 2009), cardenolide toxins, and toughest leaves among the three species (Agrawal et al., 2015). Indeed, when reared on *A. asperula* without fungicides, monarch caterpillars exhibit one of the lowest growth rates across all milkweed species (Agrawal et al., 2015). Although *A. curassavica* is a high-quality milkweed host for monarchs, its high levels of cardenolides may interact with negative effects of pesticide exposure.

In this study, we asked the following specific questions: (1) What pesticide residues are found on strawberry crops and non-target milkweeds at two small farms? (2) Are pesticide residues higher on target compared to non-target plants, early *vs.* late in the growing season, on leaves compared to flowers, and in crop fields compared to margins? (3) Do two prevalent fungicides impact monarch caterpillar feeding and growth in a dose-dependent manner in a laboratory assay? (4) To what extent are effects of fungicides on larval feeding and growth dependent on host-plant species?

## Materials & Methods

### Pesticide residue analysis

#### Collection of plant tissues at strawberry farms

In the summer of 2021, leaf and flower tissues were collected from two strawberry farms in Tompkins County, NY (Farm 1: 42.490741237293385, −76.72991435844499, Farm 2: 42.47136610627181, −76.5483703468064). Both farms were surrounded by hedgerows, other farmland, and fallow fields. According to the growers, applications of two herbicides (napropamide and terbacil) had been made at Farm 1, while applications of one herbicide (terbacil) and three fungicides (iprodione, cyprodinil, and difenoconazole) had been made to the strawberry fields at Farm 2 during the spring (March–May) of 2021.

Specific sampling sites were chosen by finding five individual patches of milkweed growing within strawberry rows on each farm and then sampling both milkweed (leaves) and strawberry tissues (leaves and flowers) among these patches. Milkweed commonly grows as a weed in and amongst small farms in New York State, and especially around perennial crops like strawberry with less cultivation than annually planted crops. These collections were made in early June and then repeated one month later, when the milkweeds were in flower (during this second visit, milkweed flowers were also collected, but strawberry flowers were absent). To address potential drift, milkweed tissues were also collected from five patches along the margins of each strawberry field during each visit (within 150 m of the in-field milkweed and strawberry plants sampled). Tissues (88 samples total) were frozen at −80 °C until extraction for pesticide residue analysis.

#### LC-MS/MS pesticide residue analysis

Tissues were extracted at the Cornell Chemical Ecology Core Facility (Ithaca, NY). Frozen leaf and flower samples of each species were extracted by modified versions of the QuEChERS procedure (Anastassiades et al., 2003) and screened for 94 (updated from 92) pesticides by liquid chromatography tandem mass spectrometry (LC-MS/MS) as in Halsch et al. (2022), Mueller et al. (2024), and Graham, McArt & Isaacs (2024). Extraction and identification protocols are as described in Halsch et al. (2022) except where noted. For the leaf samples, 2.5–5 g of material was mixed with five mL of LC-MS grade water and 10 mL of LC-MS grade acetonitrile. Samples were then homogenized and 6.5 g of salts were added, as in Halsch et al. (2022). For the flower samples, a scaled-down version of the above extraction protocol employing ∼300 mg of sample, 200 µL of LC-MS grade water, 700 µL of LC-MS grade acetonitrile, and 0.33 g of salts was used. After an additional homogenization and centrifugation (Halsch et al., 2022), one mL of supernatant (or ∼0.7 mL for the scaled-down extraction protocol) was collected and transferred to a dispersive solid phase extraction tube containing 25 mg PSA and 150 mg MgSO4. Samples were mixed, centrifuged, and 294 uL of supernatant was collected and mixed with 6 uL of an internal standard solution ((0.3 µg/mL ^13^C_6_-metalaxyl, 0.3 µg/mL ^2^H_3_-pyraclostrobin, and 0.15 µg/mL ^2^H_4_-fluopyram). Samples were then filtered (0.22 µm, PTFE) and injected onto the LC-MS system as described in Halsch et al. (2022) for detection and quantification. See Table S1 for retention times and SRM parameters for each pesticide.

### Laboratory assays

#### Plant and caterpillar rearing

During the summer of 2023, *Asclepias asperula*, *A. curassavica* and *A. syriaca* seeds were bleached (20%), nicked, and stratified for 5 days at 4 °C, then moved to a 30 °C incubator for 3 days to germinate. Seedlings were then planted in 500 mL pots with pre-moistened LM-111 All-purpose soil mix (Lambert, Riviere-Ouelle, Quebec) and grown in a growth chamber (Biochambers Inc., Winnipeg, Manitoba, Canada) with a 14 h daylength, and day: night temperature regime of 27:23 °C. Seedlings were fertilized weekly with Jacks all-purpose fertilizer (JR Peters inc., Allentown, PA, USA) (0.31 ml concentrate per liter water) and watered as needed. Plants were 4–5 weeks old at the beginning of the experiment.

Monarch butterfly (*Danaus plexippus*) eggs were obtained from Monarch Watch (Lawrence, KS, USA) on July 11 and moved to a 27 °C incubator upon arrival. Caterpillars hatched on July 13 and were placed in their individual experimental dishes within a few hours of hatching.

#### Experimental design and fungicide treatments

Each caterpillar was reared in a 1 oz solo cup, with a lid. To each cup we added a small piece of cotton (approx. 1/16 of a standard cotton ball), slightly moistened with deionized water, as well as a fresh leaf punch with the appropriate treatment applied. Punches were taken from fully developed, green leaves, from the *A. asperula*, *A. curassavica* and *A. syriaca* plants reared above.

On the first day of the experiment, each neonate caterpillar was given one standard hole punch of leaf tissue (area 0.283 cm^2^). On day two, the uneaten remainder of the leaf punch was removed from the cup and secured with clear tape onto paper for subsequent photography and area determination, and each caterpillar was given a fresh larger punch (diameter 1.11 cm, area 0.968 cm^2^), to accommodate increased feeding. This process was repeated on day 4 and day 5 for caterpillars reared on *A. curassavica* and *A. syriaca*, with one large leaf punch given each day. For *A. asperula*, leaves were too slender to accommodate the larger punches, so two standard punches were given to each caterpillar in place of one large punch, and the experiment ended one day early (day 5) due to a lack of available leaf tissue. Each individual caterpillar was serially given leaf punches treated with the same fungicide treatment throughout the experiment. At the end of the experiment (Day 5 for *A. asperula*, Day 6 for *A. curassavica* and *A. syriaca*), all remaining uneaten leaf tissue was removed and saved, and each caterpillar was weighed and then frozen at −80 °C. Saved leaf punches from each caterpillar and day where photographed and area was estimated using LeafByte (Getman-Pickering et al., 2020). Remaining leaf area was subtracted from known starting punch area to estimate total leaf area eaten per day, and these amounts were summed to estimate total leaf tissue consumption during the experiment.

For each fungicide, we chose to test a broad range of concentration, from 125–1,000 ppb, and then 10,000–300,000 ppb. The lower concentrations are representative of fungicide residues measured from field tissues in this experiment as well as previous publications on leaves, pollen, and bee bread (Mullin et al., 2010; Frazier et al., 2015; McArt et al., 2017; Graham et al., 2021; Graham, McArt & Isaacs, 2024), with the higher values chosen to represent the maximum amount of residue possible on tissues immediately after fungicide application (see below). For each fungicide, three caterpillars were tested (on each of the three milkweed species) at each concentration (see below). We note that we explicitly took a regression approach and spread our replicates across all doses tested. In other words, we had very few replicates to test differences between controls and any one fungicide dose; nonetheless, we had 24 replicates to test the slope of each fungicide’s effects on caterpillars on each plant species (and 144 replicates in total to test the main effect of fungicides on monarchs).

Each fungicide treatment consisted of only the active ingredient (cyprodinil: Chem Service, Inc. (West Chester, PA), CAS no. 121552-61-2, 99.5% purity, or difenoconazole: Millipore Sigma (Burlington, MA), CAS no. 119446-68-3, >95% purity) diluted in 95% ethanol and applied to the leaf punches. Concentrations were calculated in ppb (parts per billion) on a mass /mass (ng pesticide/g leaf tissue) basis. We estimated leaf punch mass by taking the mean mass of five fresh punches of the appropriate size for each species separately, and because these masses differed, we made up a separate set of solutions for each milkweed species. For each fungicide, we made up a stock solution for the 300,000 ppb application in 95% ethanol for each milkweed species, and then used serial dilution in 95% ethanol to achieve the following concentrations: 125, 250, 500, 1000, 10,000, 100,000, 300,000 ppb. We then pipetted the appropriate volume (2.5 uL for a standard punch or 8.5 uL for a large punch) onto the top surface of the leaf punch and used the pipet tip to immediately spread the solution evenly across the punch. Control punches received an equivalent volume of 95% ethanol, to control for any ethanol effects. For all treatments, ethanol was dried off the punches in a fume hood completely (approx. 30 mins) before they were placed in assay cups with the caterpillars.

#### Defining maximal pesticide exposure

As very few published estimates of post-spray levels of these pesticides are available from the field, we bracketed our upper levels based on field application rates of two commercial fungicides that contain both cyprodinil and difenoconazole: Vango (Atticus, Cary, NC, USA) and InspireSuper (Syngenta, Greensboro, NC, USA), and two other products that contain one of each (Switch and AproviaTop, both produced by Syngenta, Greensboro, NC, USA). These products recommend applications that result in 0.33 lbs cyprodinil and 0.114 lbs difenoconazole per acre. As strawberry is typically grown in rows and sprayed with ground equipment, we estimated that 50–75% of the active ingredient makes it onto leaves (strawberry and weeds growing among them), and then divided the amount of active ingredient applied per acre by an estimate of the number of strawberry plants grown per acre (17,500; strawberryplants.org) to estimate the amount of fungicide received per strawberry or milkweed plant (cyprodinil: 6.35 mg, difenoconazole: 2.35 mg). We estimated plant size at 4.4−8.8 g (based on exposed portion of a milkweed plant growing amidst strawberries) to obtain ranges of fungicide concentration expected: cyprodinil: 240,000–721,000 ppb, difenoconazole: 84,000–253,000 ppb. In another study of pesticide residues (Piechowicz et al., 2022), the amount of cyprodinil on apple leaves on the day of spraying was measured as 1.35 ug/cm^2^, which would be 60,000–120,000 ppb assuming leaf mass/ cm^2^ is similar to that of milkweeds. These concentrations are similar to those predicted from the T-REX model for foliar contamination resulting from field applications of cyprodinil and difenoconazole-based products (https://www.epa.gov/pesticide-science-and-assessing-pesticide-risks/models-pesticide-risk-assessment#terrestrial), which varied between 133,000–375,000 ppb for cyprodinil (Vango and AproviaTop) and 37,000–94,000 ppb for difenoconazole (InspireSuper and AproviaTop). Accordingly, we use a maximal dose of 300,000 ppb for each fungicide to determine potential effects directly after spray.

### Statistical analyses and hypothesis testing

Following the surveys of pesticide residues at the two small farms, we reduced the dataset to include only those pesticides for which we detected residues in quantifiable amounts in at least 50% of the samples (separated by farm), in one or the other month of sampling (see bold columns in Table S2). We used this filter because pesticide residue levels were, in general, very low and this approach allowed quantitative statistical comparisons of the most abundant pesticides. This left seven pesticide residues at Farm 2 (*n* = 39 samples) and two at Farm 1 (*n* = 49 samples). In the final dataset, 73% of the 371 sample-by-pesticide combinations had detectable levels of the pesticide residue.

To address our main questions and test hypotheses about where pesticide residues were most expected, data were analyzed in three two-way factorial analyses of variance using three models: (1) target crops (strawberry) *vs.* non-target milkweed, early *vs.* late in the season, (2) leaves *vs.* flowers of the two species, and (3) crop fields *vs.* margins. Separate models were run to focus on specific hypotheses with symmetrical data and allowing for tests of the interaction term between the two main effects. For example, in model 1 analyses we excluded data on flowers, since the two species did not have flowers in the same month, and we excluded samples from crop field margins, since only milkweed was available in margins. Thus, the main effects and interaction between the plant species sampled and sampling month were assessed. In model 2, leaves *vs.* flowers were compared in the same month for each species (June for strawberry and July for milkweed), allowing a specific test of pesticide residues on the two plant tissues of each species without the confounding effect of time (margins were again not considered in Model 2). We note that because the two plant species were assessed at different times (June for strawberry and July for milkweed), a significant interaction term can only be interpreted as a difference at their relevant times of being sampled, when both leaves and flowers were available for each species. Finally, in model 3, we examined only milkweed leaves in crop fields *vs.* margins across the two months of sampling. The three models were run for each pesticide separately.

For our fungicide exposure experiment with monarch caterpillars in the laboratory, we conducted linear analyses on leaf area consumed, caterpillar mass, and gross growth efficiency (mass gained per area consumed) for all data up through Day 5 with the following predictors: host plant species, fungicide identity, fungicide dose, and all interaction terms. All model terms in both sets of analyses were considered fixed effects. Residuals were examined for normality and homoscedasticity and deemed sufficiently normal for analyses. Caterpillar mortality was minimal and thus not analyzed. All analyses were conducted using JMP Pro. (version 16).

## Results

### Pesticide residue analysis

**Figure 1:**
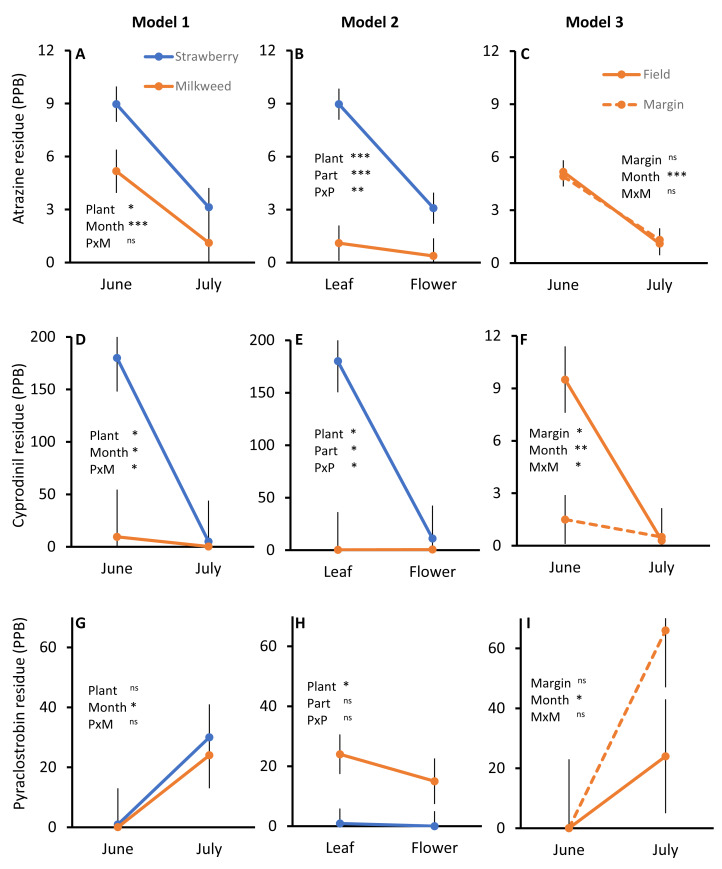
Pesticide residues on strawberry (blue) *vs.* milkweed (orange) plotted by sampling month (leaves only, Model 1, A, D, G) and tissue type (in June for strawberry and July for milkweed, Model 2, B, E, H), and residues in crop fields (solid lines) vs. margins (dashed lines) for each month (milkweeds only, Model 3, C, F, I). Results are shown for three pesticides: atrazine (A, B, C) is an herbicide, while cyprodinil (D, E, F) and pyraclostrobin (G, H, I) are fungicides (of these, only cyprodinil was applied in the year of study). Note different Y axis scales. Shown are least squares means ± SE. *P*-values are given for main effects and interaction term (full statistical analyses are given for all seven pesticides in Table S3) indicated as * (*p* < 0.05), ** (*p* < 0.01), *** (*p* < 0.001).

Across the two small farms sampled, we detected 22 pesticide residues out of the panel of 94 chemicals (Table 1, Table S2). Of these, only 13 pesticides had quantifiable levels in at least one sample, and seven met our criteria of having quantifiable levels in at least 50% of either the June or July samples from each farm. These seven systemic compounds represent four fungicides (cyprodinil, difenoconazole, fluxapyroxad, pyraclostrobin), two herbicides (atrazine and metolachlor) and one insecticide (acetamiprid). We detected quantifiable levels of all seven of these compounds on one strawberry farm (Farm 2) but only three on the other (Farm 1). We used data from both farms for the two herbicides, as these were present in >75% of all samples. Note that neither of these herbicides were applied by the focal farms in the year of study. For the other five pesticides, we only analyzed data from Farm 2 as their residues were found in just a few samples from Farm 1. Two of these five chemicals (cyprodinil and difenoconazole) were applied to the strawberry fields at Farm 2 in the study year. Key results for all three models are shown visually in Fig. 1 for atrazine, cyprodinil and pyraclostrobin. Results of statistical analyses for all seven pesticide residues are given in Table S3.

**Table 1:** Pesticide residues measured on strawberry and milkweed tissue collected from two small farms. Shown are number and percentage of positive detections and mean residue levels across all 88 leaf and flower samples collected in both fields and margins, in June and July. Results below the level of quantitation (LOQ) were converted to the sample-specific LOQ (by dividing LOQ for each compound (ng) by sample mass (g)), and means include all samples at or above the level of detection (zeros were excluded).

			Strawberry			Milkweed	
Compound	Compound type	# Positive detections	% Positive detections	Mean residue level (ppb)	# Positive detections	% Positive detections	Mean residue level (ppb)
Thiabendazole	Fungicide	1	3.03	2.02	3	5.45	1.98
Thiophanate-methyl	Fungicide	7	21.21	14.19	2	3.64	1.76
Cyprodinil	Fungicide	16	48.48	73.08	23	41.82	1.87
Azoxystrobin	Fungicide	3	9.09	0.33	0	0.00	0.00
Boscalid	Fungicide	0	0.00	0.00	1	1.82	4.83
Fluxapyroxad	Fungicide	10	30.30	12.40	14	25.45	33.57
Fluopyram	Fungicide	1	3.03	0.06	0	0.00	0.00
Difenoconazole	Fungicide	16	48.48	280.21	6	10.91	20.53
Pyraclostrobin	Fungicide	11	33.33	11.59	18	32.73	32.42
Trifloxystrobin	Fungicide	3	9.09	0.71	0	0.00	0.00
Tebuthiuron	Herbicide	19	57.58	0.38	0	0.00	0.00
Atrazine	Herbicide	32	96.97	5.42	42	76.36	3.18
Propazine	Herbicide	2	6.06	0.56	0	0.00	0.00
Napropamide	Herbicide	24	72.73	1.57	4	7.27	0.49
Metolachlor	Herbicide	29	87.88	0.87	38	69.09	0.47
Imidacloprid	Insecticide	12	36.36	6.84	1	1.82	7.70
Acetamiprid	Insecticide	5	15.15	11.06	16	29.09	38.64
Carbaryl	Insecticide	1	3.03	1.35	2	3.64	1.49
Tebufenozide	Insecticide	1	3.03	2.38	1	1.82	1.16
Spinetoram	Insecticide	1	3.03	2.16	1	1.82	10.58
Piperonyl butoxide	Synergist	1	3.03	0.23	0	0.00	0.00
Pyriproxyfen	Insecticide	4	12.12	4.97	7	12.73	0.62

Overall, we found higher pesticide residues on target strawberry compared to non-target milkweed, early *vs.* late in the growing season (model 1, Figs. 1A, 1D, 1G; Table S3), and on leaves compared to flowers (model 2, Figs. 1B, 1E, 1H; Table S3). Margins did not harbor consistently different levels of pesticide residues compared to crop fields (model 3, Figs. 1C, 1F, 1I; Table S3). Some exceptions to the general patterns suggest that drift may be leaving small amounts of pesticide residues on milkweeds. For example, the fungicides fluxapyroxad and pyraclostrobin were found more abundantly in July than June, more on milkweed than strawberry, and somewhat more so on margins than in crop fields (Fig. 1, Table S3, although this latter effect was not significant).

### Laboratory assays

**Figure 2:**
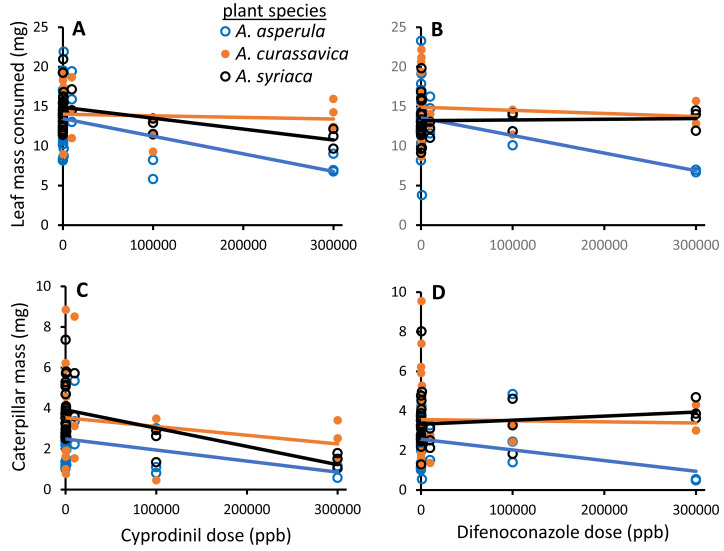
Effects of two fungicides found in our field surveys, cyprodinil and difenoconazole, on monarch caterpillars. Fungicides were tested in controlled laboratory trials at seven doses painted onto leaves of three *Asclepias* spp. milkweed host plants with monarch caterpillar feeding (A, B) and growth (C, D) over four days. Shown are the raw data. In a combined model predicting effects of the fungicides on monarch caterpillars in the laboratory, the two fungicides did not have differential effects on feeding or growth (P-values > 0.6), while both plant species and dose were highly significant for each of these metrics (P-values < 0.006, full statistical analyses are given in Table 2).

In a combined analysis of the effects of both fungicides on monarch caterpillars in the laboratory, the two fungicides did not have differential effects on monarchs, while both plant species and dose were highly significant (Table 2, Fig. 2). There were some marginal interactions between plant species, dose, and fungicide (detailed in Table 2), but overall, the strongest effects were observed on *A. asperula* and at the highest fungicide doses (those that may only be experienced directly after application). Importantly, gross growth efficiency (*i.e.,* caterpillar growth per unit of plant consumed) was also reduced by exposure to fungicides (Table 2). These results were generally mirrored in analyses of each fungicide separately (Table 2). We found reductions in caterpillar feeding, growth, and growth efficiency at high doses of cyprodinil (100,000–300,000 ppb), regardless of milkweed species. For difenoconazole, there was a detectable reduction in caterpillar feeding, but not growth or growth efficiency, caused by exposure to high doses of the fungicide on leaves (Table 2, Fig. 2).

**Table 2:** Analysis of covariance for effects of two fungicides, cyprodinil and difenoconazole, applied at various doses, on feeding and growth parameters of monarch butterfly caterpillars. Fungicide effects were analyzed in models together (“both fungicides”) and separately (below). Plant sp. indicates the three milkweed species tested and Dose indicates the continuous predictor of the eight concentrations of fungicide tested, including a no fungicide control. Significant *p*-values (<0.05) are shown in bold.

		*Consumption*		*Growth*		*Gross growth efficiency*	
	DF	F Ratio	*P*	F Ratio	*P*	F Ratio	*P*
***Both fungicides***							
Fungicide	1, 171	0.198	0.657	0.005	0.941	0.452	0.502
Plant sp	2, 171	5.359	**0.006**	12.310	**<0.001**	14.245	**<0.001**
Dose	1, 171	15.533	**<0.001**	8.508	**0.004**	5.622	**0.019**
Plant sp*dose	2, 171	4.387	**0.014**	0.434	0.649	0.429	0.652
Plant sp*fungicide	2, 171	1.204	0.302	0.239	0.788	0.017	0.984
Dose*fungicide	1, 171	0.847	0.359	3.637	0.058	4.511	**0.035**
Plant sp*dose*fungicide	2, 171	0.891	0.4123	1.652	0.195	2.440	0.090
***cyprodinil***							
Plant sp	2, 86	3.355	**0.040**	6.765	**0.002**	7.204	**0.001**
Dose	1, 86	13.791	**<0.001**	13.076	**<0.001**	12.374	**<0.001**
Plant sp*dose	2, 86	2.274	0.100	0.673	0.513	1.133	0.327
***difenoconazole***							
Plant sp	2, 85	3.179	**0.047**	5.472	**0.006**	6.642	**0.002**
Dose	1, 85	3.966	**0.049**	0.460	0.500	0.025	0.874
Plant sp*dose	2, 85	3.002	0.055	1.339	0.268	1.580	0.213

## Discussion

Recent pesticide residue analyses have revealed widespread but variable levels of agricultural chemicals in both crop and natural areas that pose a potential risk to insect pollinators and herbivores as well as other species. For example, Frazier et al. (2015) detected 53 different pesticide residues in commercial honeybees foraging on eight different crops in the USA. Brühl et al. (2021) found residues of >16 different pesticides per insect captured in natural areas adjacent to agricultural land in Germany, with the number of chemicals found being proportional to the acreage of nearby agricultural areas. In our analysis of milkweed and strawberry tissues at two small strawberry farms, we found low concentrations of pesticide residues on both plants, both within crop fields and in adjacent margins. This result is not unexpected; these farms have particularly low pesticide inputs, with their strawberry crops maintained predominantly for u-pick purposes. The most abundant pesticide residues we found were those of two systemic fungicides that had been applied to strawberry fields two months before sampling (cyprodinil and difenoconazole, up to 300 and 1,300 ppb, respectively, on strawberry foliage).

As expected, we found higher concentrations of pesticide residues in target (strawberry crop) plant tissue than in non-target (milkweed) tissue. We also saw higher residue levels in the early season (June) samples as compared to later samples (July). This pattern is consistent with residues from spring pesticide applications, or of applications from previous years that continue to degrade over time. Expectations for levels of pesticide residues on leaves *vs.* flowers also depend on the timeline of application; tissues present early in the season (*i.e.,* milkweed leaves) should contain higher concentrations of residues than later reproductive tissues (milkweed flowers). While our results for most pesticides fit these expectations, results for fluxapyroxad and pyraclostrobin showed the opposite pattern, with residues being higher on milkweeds (compared to strawberry), and late in the season, rather than early. The most likely explanation for this result is that the taller milkweed plants captured pesticide residues *via* drift coming from applications to other adjacent crops. Clearly, all potential sources of drift and contamination should be considered when assessing risk to insects dependent on non-crop plants.

While there is a current focus on measuring pesticide residues in nectar and pollen in the context of non-target effects on wild and domesticated pollinators, far fewer studies have measured pesticide residues on leaves of non-crop plants. Olaya-Arenas & Kaplan (2019) and Halsch et al. (2020), however, recently measured residues present on milkweed leaves across the agricultural landscape in Indiana, and California, respectively. These studies found a diversity of pesticide residues on milkweed leaves during the window of monarch herbivory, with the former study identifying distance from nearest cropland as a predictor of residue levels for one insecticide, thiamethoxam (Olaya-Arenas & Kaplan, 2019). For the two target fungicides in our study, cyprodinil and difenoconazole, the relatively low residue levels (up to 18 ppb and 86 ppb, respectively, on milkweed tissues; Table S2) fit with those reported in the literature; typically <<1,000 ppb (Graham et al., 2021; Rondeau et al., 2022; Siviter et al., 2023) with the highest concentrations (>2,000 ppb) being reported in apple orchards (Frazier et al., 2015; McArt et al., 2017; Mueller et al., 2024). Depending on the timing of pesticide application and insect colonization, however, we might expect monarch caterpillars to be exposed to much higher levels of these compounds while feeding on milkweeds in agricultural settings.

While we did not demonstrate effects of low doses of cyprodinil and difenoconazole on monarch caterpillar feeding, growth, and survival, we did see negative effects at high doses that could occur directly after application of these products (Fig. 2). Milkweed’s tendency to grow in and adjacent to agricultural fields (Hartzler & Buhler, 2000; Martin et al., 2021) puts it at greater risk of pesticide exposure than many other native plants. While studies of effects of fungicides on insect communities are lacking, these chemicals pose substantial risk to non-target organisms, given their generally broad modes of action (Sánchez-Bayo, 2021). Many negative effects of fungicides on non-target insects have been found, including several species of bees (Cullen et al., 2019; Belsky & Joshi, 2020), both negative and positive effects found on Colorado Potato Beetle, depending on insecticide resistance genotype (Margus et al., 2023), and negative direct and indirect effects on fungivorous and predatory mites (Pozzebon, Borgo & Duso, 2010; Bernard et al., 2010). In aquatic systems, fungicides have been shown to have direct and sublethal negative effects on aquatic invertebrates (Zubrod et al., 2019).

Although the direct mortality effects of many pesticides on insects are expected and well-known, there is growing interest in the importance of their sublethal effects in the literature (Desneux, Decourtye & Delpuech, 2007; Serrão et al., 2022; Schauer et al., 2025). For example, exposure of insects to sublethal doses of insecticides can induce stress, thus lowering immune response, as appears to be the case for bees which show higher infections of parasites after exposure to imidacloprid and thiamethoxam (Pettis et al., 2012; Alburaki et al., 2015). Chronic exposure of painted lady butterfly larvae to sublethal doses of thiamethoxam resulted in a significant reduction in adult reproduction (Schauer et al., 2025). In the case of fungicides, sublethal effects have been found for Japanese beetles, a mirid bug, and aphids, with the latter occurring as a transgenerational effect (Amarasekare & Shearer, 2013; Obear et al., 2016; Chirgwin et al., 2022), and fungicide exposure has been shown to alter the expression of stress related genes in the Colorado potato beetle (Saifullah et al., 2022). Such sublethal effects of pesticide exposure are of particular relevance to species that engage in long-distance migration, such as the monarch butterfly (Agrawal, 2017). In monarchs, larval exposure to two fungicides, azoxystrobin and trifloxystrobin, was shown to reduce adult wing length by 12.5% (Olaya-Arenas et al., 2020), which has implications for migration (Flockhart et al., 2017). Given that our experimental results were limited to the larval stage, it remains an open question whether larval exposure to cyprodinil and difenoconazole may affect traits related to flight morphology or other aspects of fitness in adult butterflies.

An important finding of this study is that direct effects of fungicides on monarch caterpillars were dependent on plant host quality. Among the three milkweed host species tested, negative effects of fungicide ingestion were strongest for caterpillars reared on *A. asperula* leaves (Fig. 2). Despite being an important host to monarchs during migration through the southern USA, this species is consistently among the poorest hosts for monarchs (Agrawal et al., 2015), and is characterized by both high concentrations of cardenolides and high potency cardenolide compounds (Agrawal & Hastings, 2019; Züst et al., 2019). Prouty et al. (2021) recently reported that negative effects of clothianidin, a neonicotinoid, on monarch pupation were also dependent on the milkweed species caterpillars were tested on. Interestingly, the authors observed the strongest negative effects on caterpillars reared on the least toxic milkweed species, while we found the opposite result. While Prouty et al. (2021) applied chemicals to whole plants, we used cut leaves in our assays, which should exclude effects mediated by plant responses to the treatment. Regardless, these findings highlight the fact that multiple environmental factors (here, plant defenses and pesticides) can combine (potentially synergize) to yield greater negative effects than either stressor would alone. More generally, poor host quality, in addition to other stressors, should be taken into account when evaluating non-target effects of pesticides (Coors & De Meester, 2008; Bray et al., 2021).

## Conclusions

Pesticides have been at the center of discussion of declining insect populations generally and the downward trend in monarch butterfly abundances in particular. Of course, there are multiple pathways of effects, ranging from direct effects of insecticides such as neonicotinoids, indirect effects of herbicides on the availability of host plants, and indirect effects of other pesticides not targeted at insects or plants (Pleasants et al., 2015; Inamine et al., 2016; Olaya-Arenas et al., 2020; Prouty et al., 2023; Deynze et al., 2024). Here we focused on milkweed plants growing in and among strawberries on small farms in New York State. Despite the fact that we sampled farms with very low pesticide use, we were able to detect residues of several agrochemicals. The extent to which such low-level pesticide residues are prevalent in the northeastern US, and implications they might have for human consumption and pollinating insects, is an important target for future work. At levels of exposure expected directly after application, we found that fungicides can have negative effects on monarch caterpillar feeding and growth, and these effects were strongest on well-defended milkweed species. Interactive effects between multiple stressors are difficult to detect and yet are thought to be common in declining populations. For monarch butterflies, while fungicide residues are unlikely to be a major driver of their population declines, fungicide sprays should be avoided in and around milkweeds during their larval period. To the extent that milkweeds in agricultural landscapes are important for monarch populations, there appear to be many overlooked additions to the list of stressors, including fungicides, which may contribute to their challenges. Additional work on pesticide residues in other parts of the monarch’s life cycle (*e.g.*, pupae and adults) as well as part of the annual migratory cycle (*e.g.*, states in the Gulf of Mexico region) would be useful for understanding additional risks.

##  Supplemental Information

10.7717/peerj.20729/supp-1 Supplemental Information 1 Retention times and optimized SRM acquisition parameters for pesticides and internal standards RT: Retention time, CE: Collision Energy.

10.7717/peerj.20729/supp-2 Supplemental Information 2 Concentration of each fungicide (Table S2a) or herbicide or insecticide (Table S2b) tested for in each sample (ppb, nanogram/gram), as measured by LC-MS/MS We show all data for the 22 chemicals that were detected in at least one sample. For samples with values highlighted in yellow, residue amounts were above the level of detection but below the level of quantification, so we report the maximal concentration (at the level of quantification) for each, as the sample-specific LOQ by dividing LOQ for each compound (ng) by sample mass (g). Agrochemical class is shown as a superscript by the name of each agent, with F=fungicide, H=herbicide, I=insecticide and *=insecticide synergist, and chemicals in bold met our criteria for statistical analysis.

10.7717/peerj.20729/supp-3 Supplemental Information 3 Results of analysis of variance testing hypotheses about higher pesticide residues in three different two-way models Model 1) on target crop vs. non-target milkweed, early (June) vs. later (July) in the growing season; Model 2) target crop vs. non-target milkweed leaves vs. flowers; and Model 3) in crop fields vs. margins for milkweed only (see Materials and Methods for hypothesis details). n=the number of samples analyzed in that model. Shown are the P values from analyses on the seven most abundant pesticide residues. Next to all P values ≤0.1, we indicate the magnitude of effect as the fold difference, e.g., “ str 2.5X” indicates that strawberry had a 2.5-fold higher residue than its comparison group (milkweed). Significant P values (<0.05) are shown in bold. Abbreviations: str=strawberry, mlk = milkweed, jun=June, jly=July, lf = leaf, fl = flower, fld = field, mr = margin. The notation for a significant interaction term in model 2 indicates the effect for one species (and no effect for the other); similarly for model 3, there is an effect in the indicated month, but not in the other.

## Additional Information and Declarations

### Field Study Permissions

The following information was supplied relating to field study approvals (*i.e.*, approving body and any reference numbers): Collection of samples was approved by Gordie Gallup of Silver Queen Farm, and Steve Cummins of Indian Creek Farm. We have chosen to refer to each farm by an assigned number, to protect farm privacy. As such, we have not listed the farm names in the methods section.

### Data Availability

The following information was supplied regarding data availability: The pesticide residue data from milkweed and strawberry tissues is available in the Supplemental File. The data on monarch caterpillar performance (growth, consumption, efficiency) on milkweed leaf discs painted with fungicide doses is available at Figshare: Agrawal, Anurag (2024). Monarch caterpillar performance (leaf mass consumed, caterpillar mass, and consumption efficiency (caterpillar mass/leaf mass consumed) on milkweed leaf discs painted with fungicide doses (ppb). figshare. Dataset. https://doi.org/10.6084/m9.figshare.27214326.v1.
