# Supplementary material for: Pesticide residues on milkweed and strawberry at small farms and non-target effects of two fungicides on monarch butterfly caterpillars
Source: PeerJ. 2026 Feb 10;14:e20729. doi: 10.7717/peerj.20729 (PMC12903895; doi:10.7717/peerj.20729)
Supplement: Supplemental Information 1 — RT: Retention time, CE: Collision Energy. [file peerj-14-20729-s001.docx]

**Supplementary Table S1**. **Retention times and optimized SRM acquisition parameters for pesticides and internal standards (RT: Retention time, CE: Collision Energy).**

| Compound | Retention Time (min) | Polarity | Precursor (m/z) | RF Lens (V) | Product (m/z) | Collision Energy (V) | Product (m/z) | Collision Energy (V) |
| --- | --- | --- | --- | --- | --- | --- | --- | --- |
| 2,4-DMPF | 5.29 | Positive | 163.0 | 89 | 107.0 | 24 | 122.0 | 16 |
| 4-Hydroxy-chlorothalonil | 10.18 | Negative | 244.8 | 141 | 174.9 | 27 | 181.9 | 29 |
| Acephate | 2.78 | Positive | 184.1 | 66 | 94.9 | 23 | 143.0 | 10 |
| Acetamiprid | 6.96 | Positive | 223.0 | 92 | 90.0 | 34 | 125.9 | 22 |
| Ametryn | 9.32 | Positive | 228.1 | 128 | 96.0 | 26 | 186.0 | 18 |
| Atrazine | 10.04 | Positive | 216.0 | 129 | 104.0 | 29 | 174.0 | 18 |
| Avermectin B1a | 17.57 | Positive | 890.3 | 176 | 305.2 | 24 | 567.3 | 13 |
| Azoxystrobin | 12.71 | Positive | 404.0 | 127 | 329.0 | 31 | 372.0 | 15 |
| Bendiocarb | 9.52 | Positive | 224.1 | 81 | 109.0 | 18 | 167.0 | 10 |
| Boscalid | 12.93 | Positive | 342.9 | 125 | 139.9 | 18 | 307.0 | 20 |
| Bromuconazole | 12.44 | Positive | 377.9 | 151 | 159.0 | 29 | 160.9 | 30 |
| Carbaryl | 10.02 | Positive | 202.0 | 73 | 127.0 | 29 | 145.0 | 10 |
| Carbofuran | 9.53 | Positive | 222.1 | 79 | 123.0 | 22 | 165.0 | 12 |
| Chlorantraniliprole | 11.56 | Positive | 483.9 | 137 | 285.8 | 11 | 452.9 | 17 |
| Chlorpyrifos | 17.01 | Positive | 349.9 | 105 | 96.9 | 31 | 197.9 | 20 |
| Clomazone | 11.39 | Positive | 240.1 | 89 | 89.0 | 47 | 124.9 | 21 |
| Clothianidin | 6.14 | Positive | 250.0 | 78 | 131.9 | 17 | 169.0 | 13 |
| Coumaphos | 15.15 | Positive | 363.0 | 127 | 227.0 | 26 | 306.9 | 18 |
| Cyanazine | 8.79 | Positive | 241.1 | 124 | 104.0 | 29 | 214.0 | 17 |
| Cyantraniliprole | 10.60 | Positive | 474.9 | 137 | 285.8 | 14 | 443.9 | 19 |
| Cyflufenamid | 15.95 | Positive | 413.1 | 128 | 241.0 | 23 | 295.0 | 15 |
| Cyprodynil | 12.35 | Positive | 226.1 | 136 | 93.0 | 35 | 108.1 | 27 |
| Cyromazine | 1.55 | Positive | 167.0 | 97 | 85.0 | 19 | 125.0 | 18 |
| Difenoconazole | 14.70 | Positive | 406.0 | 165 | 251.0 | 26 | 337.0 | 18 |
| Diflubenzuron | 13.46 | Positive | 311.0 | 100 | 141.0 | 32 | 158.0 | 13 |
| Dimoxystrobin | 13.91 | Positive | 327.1 | 89 | 116.0 | 22 | 205.1 | 10 |
| Dinotefuran | 3.70 | Positive | 203.1 | 67 | 113.1 | 10 | 129.0 | 12 |
| Diuron | 10.47 | Positive | 233.1 | 95 | 46.0 | 17 | 72.0 | 18 |
| Fenamidone | 12.78 | Positive | 312.0 | 112 | 92.0 | 24 | 236.1 | 14 |
| Fenbuconazole | 13.56 | Positive | 337.0 | 144 | 70.0 | 21 | 125.0 | 31 |
| Fenhexamid | 13.00 | Positive | 302.0 | 136 | 55.0 | 34 | 97.0 | 24 |
| Fenpyroximate | 17.13 | Positive | 422.1 | 146 | 214.1 | 30 | 366.0 | 16 |
| Fipronil | 14.61 | Negative | 434.8 | 149 | 249.9 | 27 | 330.0 | 16 |
| Fluazifop | 12.18 | Positive | 328.0 | 144 | 254.0 | 27 | 282.0 | 19 |
| Fluazinam | 16.77 | Negative | 462.8 | 138 | 397.9 | 16 | 415.9 | 20 |
| Fludioxonil | 12.37 | Negative | 246.9 | 130 | 126.0 | 32 | 180.0 | 30 |
| Flufenacet | 13.86 | Positive | 364.0 | 95 | 152.0 | 19 | 194.1 | 10 |
| Flumioxazin | 12.24 | Positive | 355.0 | 191 | 299.0 | 29 | 327.0 | 21 |
| Fluometuron | 10.05 | Positive | 233.0 | 116 | 46.0 | 18 | 72.0 | 19 |
| Fluopicolide | 13.19 | Positive | 382.9 | 131 | 144.9 | 48 | 172.8 | 24 |
| Fluopyram | 13.31 | Positive | 397.0 | 126 | 173.0 | 29 | 208.0 | 22 |
| Fluoxastrobin | 13.93 | Positive | 459.0 | 172 | 188.0 | 35 | 427.0 | 18 |
| Flupyradifurone | 7.56 | Positive | 288.9 | 110 | 89.9 | 38 | 126.0 | 19 |
| Fluxapyroxad | 12.93 | Positive | 382.0 | 133 | 342.0 | 21 | 362.0 | 15 |
| Fumagillin | 13.93 | Positive | 459.1 | 124 | 131.0 | 26 | 177.0 | 14 |
| Hexaflumuron | 15.51 | Negative | 458.7 | 118 | 275.9 | 20 | 439.0 | 10 |
| Imidacloprid | 6.45 | Positive | 256.0 | 94 | 175.0 | 18 | 209.0 | 16 |
| Indoxacarb | 16.03 | Positive | 527.9 | 164 | 150.0 | 24 | 203.0 | 39 |
| Malaoxon | 9.52 | Positive | 315.0 | 101 | 98.9 | 23 | 127.0 | 12 |
| Mandipropamid | 13.00 | Positive | 412.1 | 126 | 328.0 | 15 | 356.0 | 10 |
| Metalaxyl | 10.52 | Positive | 280.1 | 104 | 160.0 | 24 | 220.1 | 14 |
| Metazachlor | 11.11 | Positive | 278.1 | 84 | 134.1 | 22 | 210.0 | 10 |
| Metconazole | 13.74 | Positive | 320.1 | 131 | 70.0 | 24 | 125.0 | 39 |
| Methiocarb | 12.01 | Positive | 226.1 | 84 | 121.0 | 19 | 169.0 | 10 |
| Methoprotryne | 9.37 | Positive | 272.1 | 139 | 170.0 | 29 | 198.0 | 23 |
| Methoxyfenozide | 13.36 | Positive | 369.1 | 77 | 149.0 | 17 | 313.1 | 10 |
| Metobromuron | 10.79 | Positive | 258.9 | 99 | 148.0 | 16 | 169.9 | 19 |
| Metolachlor | 13.55 | Positive | 284.1 | 102 | 176.1 | 26 | 252.0 | 15 |
| Mevinphos | 6.32 | Positive | 225.0 | 82 | 127.0 | 17 | 193.0 | 10 |
| Myclobutanil | 12.78 | Positive | 289.0 | 134 | 70.0 | 20 | 125.0 | 34 |
| Napropamide | 13.13 | Positive | 272.1 | 104 | 171.0 | 19 | 199.0 | 13 |
| Oxytetracycline | 5.34 | Positive | 461.1 | 119 | 426.1 | 18 | 443.1 | 12 |
| Penthiopyrad | 14.76 | Positive | 360.1 | 126 | 256.0 | 21 | 276.0 | 15 |
| Phenmedipham | 12.08 | Positive | 301.0 | 108 | 136.0 | 20 | 168.0 | 10 |
| Phosmet | 12.46 | Positive | 318.0 | 95 | 133.0 | 36 | 159.9 | 13 |
| Picoxystrobin | 14.72 | Positive | 368.0 | 83 | 145.0 | 21 | 205.0 | 10 |
| Piperonyl butoxide | 16.46 | Positive | 356.2 | 92 | 119.0 | 34 | 177.0 | 12 |
| Profenophos | 15.94 | Positive | 374.9 | 130 | 304.8 | 19 | 346.8 | 13 |
| Prometon | 8.20 | Positive | 226.2 | 118 | 142.1 | 23 | 184.1 | 19 |
| Prometryn | 10.85 | Positive | 242.1 | 123 | 158.0 | 23 | 200.0 | 18 |
| Propazine | 11.53 | Positive | 230.1 | 130 | 146.1 | 23 | 188.1 | 18 |
| Propiconazole | 13.96 | Positive | 342.0 | 150 | 69.1 | 20 | 158.9 | 30 |
| Pyraclostrobin | 15.15 | Positive | 388.1 | 114 | 163.0 | 24 | 194.0 | 13 |
| Pyrimethanil | 9.92 | Positive | 200.1 | 150 | 82.0 | 26 | 107.0 | 24 |
| Pyriproxyfen | 16.81 | Positive | 322.1 | 118 | 96.0 | 15 | 185.0 | 21 |
| Spinetoram | 14.68 | Positive | 748.3 | 217 | 98.0 | 44 | 142.1 | 30 |
| Spinosad | 13.51 | Positive | 732.4 | 168 | 98.0 | 43 | 142.1 | 29 |
| Spirotetramat | 12.42 | Positive | 374.1 | 140 | 216.1 | 34 | 302.1 | 17 |
| Sulfentrazone | 10.20 | Negative | 384.9 | 200 | 199.0 | 36 | 307.0 | 23 |
| Sulfoxaflor | 8.10 | Negative | 275.9 | 115 | 213.0 | 17 | 261.0 | 13 |
| Tebuconazole | 13.23 | Positive | 308.1 | 130 | 70.0 | 23 | 124.9 | 37 |
| Tebufenozide | 14.26 | Positive | 353.1 | 82 | 133.0 | 19 | 297.1 | 10 |
| Tebuthiuron | 8.08 | Positive | 229.0 | 105 | 116.0 | 27 | 172.0 | 18 |
| Terbutryn | 10.97 | Positive | 242.1 | 123 | 68.0 | 40 | 186.0 | 18 |
| Tetraconazole | 13.20 | Positive | 372.0 | 152 | 70.0 | 23 | 159.0 | 31 |
| Tetramethrin | 16.64 | Positive | 332.1 | 106 | 135.1 | 18 | 164.0 | 24 |
| Thiabendazole | 4.56 | Positive | 202.0 | 130 | 131.0 | 33 | 175.0 | 26 |
| Thiacloprid | 7.88 | Positive | 253.0 | 113 | 90.0 | 36 | 125.9 | 21 |
| Thiamethoxam | 5.48 | Positive | 292.0 | 87 | 181.0 | 22 | 211.1 | 10 |
| Thiobencarb | 15.33 | Positive | 258.0 | 88 | 89.0 | 48 | 125.0 | 20 |
| Thiophanate-methyl | 9.20 | Positive | 343.0 | 119 | 151.0 | 20 | 311.0 | 10 |
| Triadimefon | 12.86 | Positive | 294.0 | 111 | 69.0 | 21 | 197.0 | 16 |
| Trifloxystrobin | 16.10 | Positive | 409.0 | 128 | 145.0 | 44 | 186.0 | 18 |
| Triflumizole | 14.50 | Positive | 346.0 | 90 | 73.1 | 16 | 278.1 | 10 |
| 13C6-Metalaxyl | 10.52 | Positive | 286.0 | 90 | 166.0 | 24 | 226.1 | 18 |
| d3-Pyraclostrobin | 15.13 | Positive | 391.0 | 105 | 167.0 | 17 | 197.0 | 10 |
| d4-Fluopyram | 13.30 | Positive | 401.0 | 139 | 177.0 | 28 | 208.0 | 21 |
