# Supplementary material for: Pesticide residues on milkweed and strawberry at small farms and non-target effects of two fungicides on monarch butterfly caterpillars
Source: PeerJ. 2026 Feb 10;14:e20729. doi: 10.7717/peerj.20729 (PMC12903895; doi:10.7717/peerj.20729)
Supplement: Supplemental Information 3 — Model 1) on target crop vs. non-target milkweed, early (June) vs. later (July) in the growing season; Model 2) target crop vs. non-target milkweed leaves vs. flowers; and Model 3) in crop fields vs. margins for milkweed only (see Materials and Methods for hypothesis details). n=the number of samples analyzed in that model. Shown are the P values from analyses on the seven most abundant pesticide residues. Next to all P values ≤0.1, we indicate the magnitude of effect as the fold difference, e.g., “ str 2.5X” indicates that strawberry had a 2.5-fold higher residue than its comparison group (milkweed). Significant P values (<0.05) are shown in bold. Abbreviations: str=strawberry, mlk = milkweed, jun=June, jly=July, lf = leaf, fl = flower, fld = field, mr = margin. The notation for a significant interaction term in model 2 indicates the effect for one species (and no effect for the other); similarly for model 3, there is an effect in the indicated month, but not in the other. [file peerj-14-20729-s003.docx]

|  |  | *Acetamiprid* | *Metolachlor* | *Atrazine* | *Cyprodinil* | *Difenoconazole* | *Fluxapyroxad* | *Pyraclostrobin* |  |
| --- | --- | --- | --- | --- | --- | --- | --- | --- | --- |
|  |  | insecticide | herbicide | herbicide | fungicide | fungicide | fungicide | fungicide | |
|  |  | n=17 | n=39 | n=39 | n=17 | n=17 | n=17 | n=17 | |
| **Model 1** | **Plant** | 0.746 | **<0.001**, str 2.5X | **<0.001**, str 2X | **0.042**, str 19X | **0.032**, str 22X | 0.588 | 0.767 | |
|  | **Month** | 0.127 | **<0.001**, jun 2.9X | **0.016**, jun 3.4X | **0.033**, jun 37X | **0.048**, jun 10X | 0.062, jly 22X | **0.033**, jly 54X | |
|  | **P x M** | 0.699 | **0.005**, str > decline | 0.866 | 0.052, str > decline | 0.074, str > decline | 0.723 | 0.831 | |
|  |  |  |  |  |  |  |  |  | |
|  |  | n=19 | n=43 | n=43 | n=19 | n=19 | n=19 | n=19 | |
| **Model 2** | **Plant** | 0.057, mlk 33X | **<0.001**, str 3X | **<0.001**, str 8X | **0.015**, str 240X | **0.018**, str >350X | **0.033** mlk 31X | **0.009** mlk 40X | |
|  | **Part** | 0.551 | 0.269 | **<0.001**, lf 3X | **0.028**, lf 16X | **0.025**, lf 31X | 0.429 | 0.453 | |
|  | **P x P** | 0.638 | 0.383 | **0.009** str lf > fl | **0.027**, str lf >> fl | **0.025**, str lf >> fl | 0.655 | 0.543 | |
|  |  |  |  |  |  |  |  |  | |
|  |  | n=17 | n=39 | n=39 | n=17 | n=17 | n=17 | n=17 | |
| **Model 3** | **Margin** | 0.215 | 0.128 | 0.165 | **0.035**, fld 5X | 0.101, fld 12X | 0.253 | 0.305 | |
|  | **Month** | 0.053, jly 29X | **<0.001**, jun 3X | **<0.001**, jun 4X | **0.009**, jun 14X | 0.057, jun >19X | **0.041**, jly 37X | **0.039**, jly >45X | |
|  | **M x M** | 0.255 | 0.685 | 0.202 | **0.028**, jun fld >> mr | 0.102, jun fld >> mr | 0.253 | 0.307 | |
|  |  |  |  |  |  |  |  |  | |

**Supplementary Table S3**. **Results of analysis of variance testing hypotheses about higher pesticide residues in three different two-way models.** Model 1) on target crop vs. non-target milkweed, early (June) vs. later (July) in the growing season; Model 2) target crop vs. non-target milkweed leaves vs. flowers; and Model 3) in crop fields vs. margins for milkweed only (see Materials and Methods for hypothesis details). n=the number of samples analyzed in that model. Shown are the P values from analyses on the seven most abundant pesticide residues. Next to all P values ≤0.1, we indicate the magnitude of effect as the fold difference, e.g., “str 2.5X” indicates that strawberry had a 2.5-fold higher residue than its comparison group (milkweed). Significant P values (<0.05) are shown in bold. Abbreviations: str=strawberry, mlk = milkweed, jun=June, jly=July, lf = leaf, fl = flower, fld = field, mr = margin. The notation for a significant interaction term in model 2 indicates the effect for one species (and no effect for the other); similarly for model 3, there is an effect in the indicated month, but not in the other.
